# A landscape review of malaria vaccine candidates in the pipeline

**DOI:** 10.1186/s40794-024-00222-3

**Published:** 2024-08-01

**Authors:** Yusuf Amuda Tajudeen, Habeebullah Jayeola Oladipo, Sodiq Inaolaji Yusuff, Samuel O. Abimbola, Muritala Abdulkadir, Iyiola Olatunji Oladunjoye, Abass Olawale Omotosho, Oluwaseyi Muyiwa Egbewande, Hameedat Damilola Shittu, Rashidat Onyinoyi Yusuf, Oluwatosin Ogundipe, Abdulbasit Opeyemi Muili, Abdullateef Opeyemi Afolabi, Salwa M. A. Dahesh, Marwa Ahmed Mahmoud Gameil, Mona Said El-Sherbini

**Affiliations:** 1 Department of Microbiology, Faculty of Life Sciences, University of Ilorin, P.M.B. 1515, Ilorin, 240003 Nigeria; 2 Department of Epidemiology and Medical Statistics, Faculty of Public Health, College of Medicine, University of Ibadan, P.M.B 5017 G.P.O, Ibadan, Oyo State Nigeria; 3 Faculty of Pharmaceutical Sciences, University of Ilorin, P.M.B. 1515, Ilorin, 240003 Nigeria; 4 Department of Medicine, Faculty of Clinical Sciences, Obafemi Awolowo University, Ibadan- Ife Rd, Ife, 220282 Osun State Nigeria; 5 Cyprus International Institute of Environmental and Public Health, Cyprus University of Technology, Limassol, 3036 Cyprus; 6 Department of Microbiology, Faculty of Pure and Applied Sciences, Kwara State University, P.M.B 1530, Malete-Ilorin, Ilorin, Nigeria; 7 Faculty of Basic Medical Sciences, Ladoke Akintola University of Technology, P.M.B 4000, Ogbomosho, Oyo State Nigeria; 8 Faculty of Biomedical Sciences, Department of Microbiology and Immunology, Kampala International University, Bushenyi, Uganda; 9 Research Institute of Medical Entomology, General Organization for Teaching Hospitals and Institutes, GOTHI, Damietta, Egypt; 10 Department of Medical Parasitology, Faculty of Medicine, Damietta University, Damietta, Egypt; 11 Department of Medical Parasitology, Faculty of Medicine, Cairo University, Cairo, 11562 Egypt

**Keywords:** Malaria vaccine, Vaccine technologies, Global health, Vaccine-induced immune response, Clinical trials

## Abstract

**Background:**

Globally, malaria continues to pose a major health challenge, with approximately 247 million cases of the illness and 627,000 deaths reported in 2021. However, the threat is particularly pronounced in sub-Saharan African countries, where pregnant women and children under the age of five face heightened vulnerability to the disease. As a result, the imperative to develop malaria vaccines especially for these vulnerable populations, remains crucial in the pursuit of malaria eradication. However, despite decades of research, effective vaccine development faces technical challenges, including the rapid spread of drug-resistant parasite strains, the complex parasite lifecycle, the development of liver hypnozoites with potential for relapse, and evasion of the host immune system. This review aims to discuss the different malaria vaccine candidates in the pipeline, highlighting different approaches used for adjuvating these candidates, their benefits, and outcomes, and summarizing the progress of these vaccine candidates under development.

**Method:**

A comprehensive web-based search for peer-reviewed journal articles published in SCOPUS, MEDLINE (via PubMed), Science Direct, WHO, and Advanced Google Scholar databases was conducted from 1990 to May 2022. Context-specific keywords such as “Malaria”, “Malaria Vaccine”, “Malaria Vaccine Candidates”, “Vaccine Development”, “Vaccine Safety”, “Clinical Trials”, “mRNA Vaccines”, “Viral Vector Vaccines”, “Protein-based Vaccines”, “Subunit Vaccines”, “Vaccine Adjuvants”, “Vaccine-induced Immune Responses”, and “Immunogenicity” were emphatically considered. Articles not directly related to malaria vaccine candidates in preclinical and clinical stages of development were excluded.

**Results:**

Various approaches have been studied for malaria vaccine development, targeting different parasite lifecycle stages, including the pre-erythrocytic, erythrocytic, and sexual stages. The RTS, S/AS01 vaccine, the first human parasite vaccine reaching WHO-listed authority maturity level 4, has demonstrated efficacy in preventing clinical malaria in African children. However, progress was slow in introducing other safe, and feasible malaria vaccines through clinical trials . Recent studies highlight the potential effectiveness of combining pre-erythrocytic and blood-stage vaccines, along with the advantages of mRNA vaccines for prophylaxis and treatment, and nonstructural vaccines for large-scale production.

**Conclusion:**

Malaria vaccine candidates targeting different lifecycle stages of the parasite range from chemoprophylaxis vaccination to cross-species immune protection. The use of a multi-antigen, multi-stage combinational vaccine is therefore essential in the context of global health. This demands careful understanding and critical consideration of the long-term multi-faceted interplay of immune interference, co-dominance, complementary immune response, molecular targets, and adjuvants affecting the overall vaccine-induced immune response. Despite challenges, advancements in clinical trials and vaccination technology offer promising possibilities for novel approaches in malaria vaccine development.

## Introduction

Malaria, a protozoal infection caused by various *Plasmodium* parasite species and transmitted by female *Anopheles* mosquitoes carrying the infective stage, remains a critical global health concern. Among the five species known to infect humans (*P. falciparum, P. vivax, P. malariae, P. ovale*, and *P. knowlesi), P. falciparum* and *P. vivax* are the most prevalent and responsible for the majority of malaria case [1–3]. The disease is endemic in 87 countries, particularly in the tropical and subtropical regions. According to the World Health Organization (WHO) malaria report in 2021, Africa accounted for 95% of the estimated 241 million cases and 627,000 malaria deaths worldwide [3,4]. 

 Malaria transmission dynamics are intricately linked to environmental and socioeconomic factors within communities [5–7]. Despite continuous efforts to reduce the disease burden and fatalities, the goal of malaria elimination is still a significant challenge [3]. v Vaccines for malaria prevention is an important area of research targeting different life cycle stages of the parasite. These vaccines incorporate antigens expressed during pre-erythrocytic, erythrocytic, and/or mosquito sexual stages to mitigate or halt the spread of the parasite in the community [5]. 

Malaria vaccine development has made significant progress, with the first human anti-parasite vaccine successfully passing regulatory scrutiny [8]. This milestone marked the first human parasite vaccine to attain the highest level of regulatory scrutiny, known as WHO-listed authority maturity level 4 (WLA ML4) [9]. 

Simultaneously, clinical trials for novel malaria vaccine candidates have rapidly advanced. Some of these vaccine candidates aim to leverage upon the efficacy of the RTS, S/AS01 vaccine in preventing clinical malaria in African children, while others aim to protect pregnant women from malaria. In addition, these vaccines also interrupt the parasite transmission cycle, by blocking *P. falciparum* infection or transmission to mosquitoes [9]. Over the last two decades, the number of new malaria vaccine trials registered on ClinicalTrials.gov, a prominent platform for clinical trials registration since 2000, has remained steady at approximately ten trials per year [9]. In this comprehensive review, we classified malaria vaccine candidates according to the parasite lifecycle and highlighted the progress and limitations of several vaccine candidates in the development pipeline.

## Methodology

A comprehensive narrative review of the literature published in English from 1990 to May 2022 was undertaken. The conducted search involved relevant databases and conventional search engines, incorporating information from SCOPUS and MEDLINE (via PubMed), Science Direct, WHO, and Advanced Google Scholar. The search protocol utilized a combination of keywords related to malaria, vaccine candidates, and vaccine development. Subsequently, the search was refined with a focus on context-specific keywords such as “Malaria”, “Malaria Vaccine”, “Malaria Vaccine Candidates”, “Vaccine Development”, “Vaccine Safety”, “Clinical Trials”, “mRNA Vaccines”, “Viral Vector Vaccines”, “Protein-based Vaccines”, “Subunit Vaccines”, “Vaccine Adjuvants”, and “Vaccine-induced Immune Responses”, and “Immunogenicity”.

The reference lists of retrieved articles were thoroughly examined to identify additional titles for potential inclusion in the review. Exclusion criteria were applied, excluding articles on malaria vaccines and vaccine candidates published in languages other than English, those unrelated to malaria or vaccine candidates in preclinical and clinical stages of development, articles lacking sufficient data on malaria vaccine candidates, and correspondence articles. In alignment with the predefined eligibility criteria for this review, which focuses on peer-reviewed articles published in English providing detailed information on malaria vaccine candidates, their development, and evaluation, the final selection of the articles was reviewed and incorporated into our study.

## Classification of malaria vaccine candidates

Potential malaria vaccines are typically categorized based on the stage of the malaria parasite’s life cycle at which they elicit immunity. Currently, there are three classes of vaccines: Pre-erythrocytic vaccines refer to vaccines targeting the liver stage of the parasite  (including e.g. genetically attenuated sporozoites, irradiated sporozoites, CSP-based subunit vaccines, etc.); erythrocytic vaccines refer to vaccines targeting the blood stage of the parasite (subunit vaccines (e.g. AMA-1, MSP-1 etc.) including pregnancy-related vaccine targeting e.g. VARCSA; transmission-blocking vaccines refer to vaccines targeting the gametocyte and the mosquito stages of the parasite. Thus, different antigens and stages of the parasite’s asexual and sexual life cycle can be targeted by the vaccine candidate.

## Pre-erythrocytic vaccines

Pre-erythrocytic vaccines, also known as liver-stage/ anti-infective vaccines, focus on the parasite sporozoites by inducing specific antibodies that can destroy infected hepatocytes or inhibit the proliferation of sporozoites (spz) within liver cells. This prevents the release of merozoites into the bloodstream, thereby preventing the clinical manifestations of malaria in infected individuals [10]. Pre-erythrocytic vaccines are further classified into whole sporozoite vaccines (WSV) and subunit vaccines. The primary focus of WSV research has been attenuated sporozoite vaccines, while subunit vaccines involve the use of sporozoite recombinant proteins and DNA or viral vectored proteins [10]. These viral vectors can enhance the production of reactive CD4^+^ and CD8^+^ T-lymphocyte (T cells) and elevate antibody (Abs) titers against malaria parasites, resulting in strong immune response.

### Whole sporozoite vaccines (WSV)

#### Description

The whole sporozoite vaccines (WSV) currently in development are radiation attenuated Spz (RAS), Spz administered under drug coverage , and genetically attenuated parasite Spz (GAP) vaccines [11]. WSVs offer a broader range of antigens, allowing them to act on both extracellular sporozoite and intracellular hepatic stage of the malaria parasite life cycle, in contrast to subunit vaccines that only target the intracellular stage [12]. 

Live-attenuated sporozoites infect liver cells but do not cause blood-stage infection. RAS and GAP vaccines arrest in liver stage development, while CPS vaccines are cleared during the early phases of red blood cell infection [12]. Consequently, CPS or WSV can induce cellular immune response reducing the hepatic stage-parasite burden and consecutively, reducing the chance of merozoites erythrocytic invasion in the blood stream [12]. 

#### Mechanism of action

Compared to the ‘natural’ immunity developed by individuals living in endemic regions, whole sporozoite vaccination aims to induce strong and sterilizing immune responses [13,14]. Natural transmission of malaria introduces only a few doses of sporozoite antigens, resulting in a weak or no immune response. However, vaccination with high doses of whole sporozoites induces robust CD8^+^ and CD4^+^ responses [15,16]. The first site of antigen engagement and priming of parasite-specific CD8^+^ T cells occurs in the skin-draining lymph nodes near the inoculation site [17]. Dendritic cells cultivated with sporozoites have been shown to present parasite antigens to CD8^+^ cells, indicating that protective CD8^+^ T cells are primarily primed in the lymph node draining the vaccination site [17,18] CD8^+^ T cells present in the liver play a crucial role in the rapid elimination of the parasite during liver stage replication of the parasite [19]. However, the specific mechanisms employed by CD8^+^ T cells to eliminate hepatic-stage parasites are not well understood. Studies demonstrated that within four hours of sporozoite challenge in situ, more than 50% of antigen-specific cells started producing IFN-γ, indicating that liver tissue-resident CD8^+^ T cells can respond swiftly to live sporozoite challenge, which is crucial in the protection against liver-stage malaria. Evidently, liver tissue-resident CD8^+^ T-cell mediated protection are considered at the forefront , with close contact and cognate peptide recognition between the effector T cell and infected hepatocyte protection against the pre-erythrocytic stages of malaria [17,19]. According to Medica and Sinnis, the major challenge in eliminating hepatic stage parasites during the natural transmission is its “quiet” nature as very few sporozoites (about 10–100 sporozoites/mosquito) are inoculated with fewer infected liver cells as a result [20]. Fortunately, this creates a golden opportunity to target hepatic parasitic stages due to their intracellular existence in small numbers and presence in a cell expressing the class 1 major histocompatibility complex (MHC). Thus, WSV works by amplifying the number of parasites inoculated, thereby increasing ‘silent’ infection to a complete immunological insult to induce protective immunity [17]. 

#### Challenges and benefits of WSV

One of the primary advantages of whole sporozoite vaccines (WSVs) compared to sub-unit vaccines is their ability to elicit an immune response against pre-erythrocytic stages of the malaria parasite life cycle, potentially leading to vaccine-induced immunity. The sporozoites administered under drug coverage can be considered as chemoprophylaxis vaccination while radiation-attenuated sporozoites which have demonstrated over 90% protection against malaria are largely in clinical trials to date [10]. However, there are challenges associated with the manufacturing of adequate quantities of sporozoites on a large scale. Among the methods of sporozoite vaccination is direct inoculation through mosquito bites or injections after complex and tedious mosquito dissections [12]. Current methods of vaccination include the development of human-infective *P. falciparum* sporozoites demonstrating for the first time 60–70% protection when the same parasites are administered via intramuscular (I.M.) route [12]. 

### Subunit blood-stage vaccines

#### Description

Subunit vaccines are based on the immunodominance of the circumsporozoite protein, which has been observed to confer full protection in the whole sporozoite vaccines (WSVs) [21]. The CS protein (CSP) has thus been the primary focus of research in developing immunity through subunit vaccines and recombinant bacterial and viral vectored vaccines [22,23]. An example of a subunit vaccine is RTS, S recently approved by the WHO, in which the *Plasmodium falciparum* CS subunits are expressed within immunogenic Hepatitis B surface antigen [17]. Other examples include the R21 vaccine and viral and bacterial vectored vaccines, which also incorporate proteins such as thrombospondin-related adhesion protein (TRAP) and liver stage antigen (LSA) [10]. 

#### Mechanism of action

Subunit vaccines contain protein or glycoprotein part of a pathogen that can elicit a protective immune response. According to Mehreen Datoo et al., both R21 and RTS, S share a similar structure, with Hepatitis B surface antigen (HBsAg) fused to their C-terminus and central repeats of the CSP [24]. However, R21 lacks the excess of HBsAg present in RTS, S, resulting in a higher density of CSP epitopes and the generation of higher levels of malaria-specific anti-Asn-Ala-Asn-Pro (NANP) antibodies. While R21 exclusively consists of fusion protein moieties, RTS, S comprises only 20% of CSP and 80% of HBsAg monomers, leading to reduced CSP coverage on the viral-like particle (VLP) surface [25,26]. Another distinction between the two vaccine candidates also lies in their adjuvants. While RTS, S is often administered with adjuvant AS01 or AS02, R21 employs Matrix-M (MM) as its adjuvant, a saponin-based adjuvant with strong immunogenicity capable of inducing both humoral and cellular responses. Consequently, R21 demonstrates higher efficacy at 77% compared to RTS, S, which achieves 56% at 12 months post- vaccination and 33% at 18 months, though the latter has been tested thoroughly. However, only RTS, S has received approval for use in children residing in malaria-endemic regions following a successful phase 3 clinical trials, while R21 is currently in the phase 2 clinical trials [24]. 

#### Challenges and benefits

One significant advantage of subunit vaccines, specially the RTS, S/AS01, and R21/MM vaccines is their potential for commercial production and cost-effectiveness in the distribution of the vaccine. This is particularly crucial in the global efforts to improve malaria control and sustainably eliminate the disease, considering its prevalence in resource-constrained regions [24]. However, a major challenge associated with these vaccines is their reduced efficacy compared to the level of sterile, strain-transcending immunity provided by radiation-attenuated sporozoites [17]. Another major challenge is the observed waning immunity over time with the RTS, S vaccine, with efficacy dropping as low as 30% at 18 months post-vaccination. In contrast, the R21/Matrix M vaccine has shown a more stable immune response, as the vaccine efficacy observed at 6 months after immunization remains comparable to that seen at 12 months in a randomized controlled trial conducted in Burkina Faso [24,27]. 

### Erythrocytic/blood-stage vaccines

#### Description

The asexual stages of *Plasmodium*, which involves repeated replication cycles within erythrocytes, represent desirable targets for malaria vaccine development. The emergence of strain-specific immunity due to polymorphism has consistently resulted in diminished vaccine efficacy. Consequently, the formulation of vaccines utilizing conserved and naturally acquired antigens holds promise for achieving heightened efficacy [28]. *Plasmodium falciparum* Reticulocyte-binding Protein Homolog 5 (PfRipr) stands out as a novel vaccine specifically designed for the asexual blood stage, aiming to elicit potent growth inhibitory antibodies. The protein complex of PfRipr/PfCyRPA/Rh5 is recognized as a promising candidate for vaccine development, and its combination with adjuvants enhances its suitability [28]. Examples of invasion molecules currently in phase 2 of clinical development against the asexual blood stages include Rh5.1/AS01 and ChAd63.MVARh5. Additionally, ongoing developmental efforts target VAR2CSA with vaccines such as PRIMVAC and PAMVAC, with a specific focus on examining its efficacy on malaria during pregnancy [28]. 

#### Mechanism of action

During the liver stage, a vaccine targeting the asexual stage induces IFN-γ synthesis by activating CD8 + T cells, leading to the production of potent antiparasitic nitric oxide (NO) by induced infected hepatocytes. Other mechanisms of action during the liver stage include the activation of apoptosis of infected hepatocytes and the recognition of parasite antigens by natural killer (NK) cells [25,26]. Significant data have been accumulated for LSA and other antigens expressed by liver-stage parasites. LSA-1, specifically expressed by the parasite without homologs in mice and nonhuman primates, makes LSA-1-containing vaccines potential candidates for clinical trials [28]. 

Merozoites burst from infected cells, making them vulnerable to circulating antibodies. However, they remain free for a transient period and thus can be a target for the development of anti-merozoite vaccines. One of the mechanisms studied in the merozoite stage is the blocking of their attachment site, invasion, or development inside the RBCs. In vitro analysis has shown that Duffy binding protein (DBP) and erythrocyte binding antigen (EBA-175) act as receptors in the RBC for *P. vivax* and *P. Falciparum*, respectively. Blocking these receptors can thus prevent parasite invasion. The antibodies inhibit the parasites from binding to the Duffy Antigen/Receptor for Chemokines (DARC) receptor by receptor blocking. The DARC receptor is significant in the context of malaria infection because it serves as an entry point for the parasites into red blood cells. If the parasites are unable to bind to the DARC receptor due to the presence of these inhibitory antibodies, it disrupts the initial step of the infection process. This interference with the binding process can be instrumental in preventing or reducing the severity of the infection. A recent study observed the inhibition of plasmodial growth in vivo and in vitro by eliciting antibodies against merozoite surface protein (MSP-1) [24]. A blood-stage vaccine is effective in reducing clinical illnesses, inducing sterile immunity, and reducing transmission in animal models by controlling parasite density and decreasing ultimate gametocytes in the bloodstream. Therefore, an effective blood-stage vaccine would serve dual purposes for treating malaria and preventing clinical illnesses [29,30]. 

Infected RBCs express parasitic antigens and asexual stage vaccines act on these infected RBCs through mechanisms such as the release of cytokines by CD4 + T cells to exert parasiticidal and parasitostatic effects. Additionally, activation of macrophages occurs, and antibodies against expressed molecules induce the complement system cascade or opsonization by monocytes. Antibodies produced against parasitized RBCs enable phagocytes to prevent the development of cerebral malaria. Ring erythrocyte surface antigen (RESA) has been a potential multi-antigen used with MSP-1 and 2 in a clinical trial [28]. Finally, parasite toxins have been targeted for vaccine development, with tumor necrosis factor-alpha (TNF-α) being a major toxin.

#### Challenges and benefits

Asexual blood stage vaccine has a constrained timeframe for the neutralization of antibodies targeting merozoite antigens. Merozoites are the invasive form of the parasite, and the timely neutralization of antibodies is crucial for preventing the invasion of red blood cells and the progression of the malaria infection. The limited window of opportunity for antibody action underscores the need for precise vaccine design and rapid immune response [29]. In the context of malaria vaccines targeting clinical symptoms, those directed against TNF-α (tumor necrosis factor-alpha) show promise in reducing malaria-related symptoms such as fever. However, it is crucial to administer these vaccines in combination with other anti-malarial chemotherapies. Using a vaccine solely targeting clinical disease without simultaneously clearing the parasite may have adverse effects on malaria relapse, especially in non-immune individuals with chronic disease [30]. Other blood-stage vaccine candidates include PfCSP (*Plasmodium falciparum* Circumsporozoite Protein) and PvCSP (*Plasmodium vivax* Circumsporozoite Protein). While PfCSP has been extensively studied, significant challenges arise with polymorphic variants of PvCSP, such as VK210 and VK247. The presence of these variants can impact the efficacy of CSP-based vaccines, necessitating careful consideration of the parasite species. Another illustrative example is the comparison between Pfs230 and Pvs230. Understanding the differences between these antigens and their respective roles in the pathogenicity of *Plasmodium falciparum* and *Plasmodium vivax* is essential for developing vaccines that target multiple stages of the parasites, thereby enhancing overall efficacy [29]. Thus, the challenges related to antigen selection, merozoites invasion pattern, and timely antigen neutralization are crucial for the development of effective asexual blood-stage malaria vaccines. Additionally, considerations about the complexities associated with specific antigens like PfCSP, PvCSP variants, and other proteins such as Pfs230 and Pvs230 contribute to the nuanced landscape of malaria vaccine research [31]. 

### Sexual stage vaccines

#### Description

Sexual stage vaccines, also known as transmission-blocking vaccines (TBVs), target the stages of the malaria parasite-life cycle that occurs in infect mosquito vectors, effectively preventing the spread of malaria within the community [31]. The complete life cycle of the malaria parasite involves both the mosquito vector, where sexual reproduction takes place, and the human host, where significant asexual replication occurs in the hepatocytes and red blood cells [32]. 

#### Mechanism of action

Among the various stages of the parasite’s life cycle, blocking sexual development in the mosquito midgut appears to be the most effective strategy for inhibiting malaria transmission [32]. Mosquito-stage transmission-blocking vaccines (TBVs) stimulate the production of antibodies in the bloodstream. These antibodies, when mosquitoes ingest blood meals containing the vaccine-induced antibodies, can modify the viability of the parasite, inhibit their development, or disrupt their interaction in the mosquito midgut. This mechanism ultimately reduces or blocks malaria transmission from mosquitoes to human hosts [32]. 

Among the ongoing projects in preclinical development for sexual-stage vaccines, two of the most promising candidates are based on the Pfs25 antigen [33]. Consequently, the most advanced and currently the only candidates in the TBV pipeline are recombinant vaccines that utilize the Pfs25-based antigen [32]. 

The development of transmission-blocking vaccines (TBVs) that specifically target the sexual stage parasites is considered a crucial component in the global efforts to eradicate malaria [32]. However, in the field of vaccine development, only a limited number of vaccine candidates have undergone thorough evaluation as potential TBV targets. These candidates include the parasite surface proteins P230, P48/45, P25, and P28, and the mosquito target AnAPN1 [32]. Targeting components of the mosquito midgut in vaccines is of particular interest because it has the potential to reduce the vector’s competence and simultaneously block the transmission of multiple malarial species. Promising early proof-of-concept evidence has shown that antibodies induced by P230, P48/45, P25, and P28 can confer transmission-blocking activities [32]. 

#### Challenges and benefits

TBVs offer a significant benefit by reducing the spread of resistant parasites [31]. Although TBVs do not provide direct protection to vaccinated individuals, they contribute to the overall protection of the community at large. However, the production of recombinant TBVs poses substantial challenges. Parasite surface proteins such as P230, P48/45, P25, and P28 have unique structural characteristics rich in cysteine residues, which impose limitations on the expression methods used to produce recombinant products that maintain their native conformation [32]. For instance, early attempts to produce recombinant Pfs25 in *Escherichia coli* – a commonly used bacteria for gene expression, resulted in products that were not recognized by transmission-blocking monoclonal antibodies (mAbs). Furthermore, antibodies induced by this recombinant Pfs25 failed to block transmission [7]. 

The selection of suitable adjuvants also presents challenges in TBV formulation. Adjuvants intended for use in TBV formulation must possess a broad safety profile in humans in addition to immune-boosting properties. This is because TBVs typically require higher vaccination coverage to achieve herd immunity [32]. However, the conservative approach taken in selecting adjuvants for TBV formulation limits the available options due to the lack of durable and efficient immune response, another approach involved the use of nanoparticle combination. Moreover, the benefits provided by TBVs are delayed for those who receive them since the vaccine does not immediately reduce the risk of malaria infection. Protection is achieved through herd immunity, which takes time to develop [32]. 

Furthermore, the development of TBVs, similar to other malaria vaccines, has been slow due to a weak economic interest [31]. TBVs have faced skepticism and confusion, leading to a lack of commercial interest in their development, with some believing that TBVs will have limited impact [31]. Consequently, there is insufficient funding available for TBV research and development.

### Multi-stage vaccine preparations (pre-erythrocytic, erythrocytic and transmission-blocking vaccines)

#### Components

A multistage vaccine represents a novel approach to target all stages of parasite development and thus achieve sterile protection, while also targeting a reduction in transmission. Typically, a multi-antigen formulation is employed in the development of a multistage vaccine, aiming to target different stages of *Plasmodium* life cycles. Such a vaccine provides protection and transmission-blocking (TB) benefits, thereby reducing the overall disease burden. WHO set the development of a multistage vaccine against clinical disease and TB as a strategic goal in 2013, to be achieved by 2030 [35,36].

In the study conducted by Keyes et al., the protective and TB effect of an improved RTS, S/AS01 vaccine were investigated by either mixing the vaccine with Pfs25-IMX313/AS01 as a single formulation or co-administering them. Both vaccines demonstrated a stimulating effect against PfCSP and Pfs25, respectively, compared to that observed with single-antigen vaccines. Furthermore, a multistage AdHu5-AAv1 Pfs25-PfCSP vaccine, aimed at reducing vaccination cost, exhibited potential efficacy against transgenic *P. berghei* in mice, comparable to a single-antigen formulation.

Additionally, the use of multiple antigen protein (MAP) containing a series of T- and B-cell epitopes in conjunction with alum adjuvant, induced low immunity but significantly high antibody titer in monkeys infected with sporozoites. This suggests that the vaccine could be a suitable candidate for individuals with a history of malaria infection. Moreover, the production of a male and female gametocyte stage is derived from asexual forms (merozoites). Therefore, a reduction in asexual parasite levels (hepatic and erythrocytic phases) in vaccinated individuals may decrease the occurrence and volume of gametocytes due to the presence of anti-glutamate-rich protein (GLURP) antibodies. The GLURP protein acts synergistically by targeting both the sexual and asexual stages, preventing mutation, and blocking transmission by gametocytes [37,38]. 

During the sexual stage of *Plasmodium*, the antigen Pfs48/45 is involved in gamete fertilization, making it a potential target for transmission-blocking. Expression of this antigen by gametocytes in the human host leads to the development of acquired anti-Pfs48/45 antibodies, which confers resistance to transmission. A functional folded fragment of Pfs48/45 (10 C) fused with GLURP (GLURP, RO) has been combined to form a multi-stage chimera vaccine, inducing antibodies with both transmission-blocking and asexual stage activity through antibody-dependent cellular inhibition against *Plasmodium falciparum* strain [37]. 

### New and old technologies used in the development of malaria vaccines

The development of highly efficient and durable vaccines against the human malaria parasite remains a key priority [39]. To achieve this goal of next-generation vaccines, it is crucial to leverage the successes of current pre-erythrocytic subunit and whole sporozoite-based vaccines, as well as to explore new strategies that incorporate blood-stage or transmission-blocking immunity [39]. Recent advancements in high-throughput biology and computation have greatly enhanced our understanding and ability to design effective malaria vaccines [40]. 

Several novel approaches have emerged in the field of malaria vaccine development, including mRNA vaccinology, nanotechnology, vaccine combination [41], reverse vaccinology, and structural vaccinology [40]. In this section, a concise overview will be provided for the remaining novel approaches, while detailed explanations of the mRNA vaccine, nanotechnology, and viral vectored vaccine will be presented in a subsequent sub-section of the study.

Vaccine combination refers to the strategy of combining a pre-erythrocytic stage vaccine with another vaccine that induces an immune response against the blood stage of the malaria parasite. This approach aims to address the challenges associated with blood-stage infection of malaria [42,43]. By targeting multiple stages of the parasite’s life cycle, vaccine combinations have the potential to enhance protection and provide a more comprehensive defense against malaria.

#### mRNA vaccine

The mRNA vaccine is an innovative approach to vaccinology that enables vulnerable host cells to express antigens, transmembrane proteins, and viral glycoproteins with a natural glycosylation profile [44]. There are two types of mRNA vaccines currently available: self-amplifying mRNA and conventional mRNA [45]. Unlike traditional vaccines which trigger an immune response for antibody production, the mRNA vaccine delivers the antigenic sequence to cells, enabling them to express the encoded protein and present it to the immune system [44]. Upon introduction into the body of an infected malaria patient, the mRNA in the vaccine is recognized by endosomal or cytosolic receptors, leading to the activation of the Type I interferon pathway and the production of chemokines and pro-inflammatory cytokines. These signaling molecules activate antigen-presenting cells and stimulate a robust adaptive immune response against the pathogen, resulting in full-fledged immunity against malaria infection [46]. 

Moreover, mRNA offers several advantages as a new approach to malaria treatment. It allows the delivery of a more complex, multi-antigen vaccine by combining sequence variants and targets, thereby enhancing immunity against diseases like malaria [47]. Additionally, RNA-based vaccines can be utilized to target *Plasmodium* macrophage migration inhibitory factor (PMIF), a parasite antigen that attenuates the host’s T cell responses during pre-erythrocytic or erythrocytic stages of infection. PMIF protects against *Plasmodium* infection at the liver and blood stages, providing complete protection from re-infection [48]. A study demonstrated that mice immunized with PMIF exhibited robust cellular and humoral immune responses [49]. 

However, the distribution and efficacy of mRNA vaccines in treating malaria and other diseases face certain challenges, such as the availability of raw materials, delivery efficiency, cell targeting, material safety, route of administration, and vaccine thermostability [50]. The supply of raw materials, including plasmid DNA templates and deoxynucleotide triphosphates, and in vitro transcription, is limited globally, leading to increased costs [51]. Delivery efficiency is another significant challenge as the naked form of the mRNA is easily recognized by the immune system and rapidly degraded by nucleases after entering the body, which reduces the vaccine’s efficacy [52]. 

#### Nanotechnology

Nanotechnology is an innovative tool, that offers both short-term and long-term solutions for the treatment and vaccination of malaria [53]. In the context of vaccines, various nanostructures have been developed and tested to generate and modulate the immune response. These nanostructures serve as delivery platforms, incorporating a cocktail of antigens (or DNA encoding for antigens), adjuvants, and immunomodulatory molecules [54–56]. One example of a nano-vaccine is the first malaria vaccine Mosquirix, RTS, S which provides partial protection against malaria in young children [57,58]. Recent research has shown the efficacy of a multistage, multiantigen vaccine in mice and rabbits through the spontaneous nanoliposome antigen particularization [59], indicating that nanotechnology can provide affordable large-scale production of successful malaria vaccines and offer new approaches to protect individuals of all ages [57]. 

Another nanotechnology vaccination approach is the use of viral envelopes, known as virosomes, for targeted delivery of incorporated antigens. This approach has successfully induced the production of parasite growth-inhibitory antibodies against the apical membrane antigen 1 (AMA1) of blood-stage malaria parasites [60]. Currently, clinical trials are underway for self-assembled nanoparticles based on CSP epitopes [61]. DNA vaccination, which involves the use of DNA encoding for certain antigens, is another promising approach, but the optimal route of administration and carrier for DNA vaccines is still being explored. Nanocarriers can protect and deliver DNA, overcoming the low stability of free DNA in biological fluids and promoting cellular uptake [54]. 

Designing an effective subunit protein vaccine for malaria is challenging due to the difficulty in selecting and prioritizing the antigens or combinations of antigens that can induce a maximal protective response. To eradicate malaria, a nanotechnological approach is being developed to provide a ‘mixed drug’ and ‘vaccine-like’ activity, targeting *Plasmodium* merozoite invasion into RBCs as the drug activity, and fine-tuning the immune response against extracellular parasites by targeting the merozoite– nanoparticle complex in immune cells to potentially induce protective immunity (‘vaccine-like activity) [53]. This approach involves creating nanostructures (nanomimics) based on polymer vesicles (polymersomes) that are functionalized with a molecule found on red blood cells. These nano mimics bind to *P. falciparum* merozoites after egress from RBCs and inhibit subsequent invasion. Strong multivalent interactions between nano mimics and parasites in vitro have demonstrated highly potent inhibition of invasion by these nanostructures [53]. 

The potential impact of nanotechnological approaches includes the formulation of single, cost-effective vaccines with increased efficacy and innovative combinations for eliminating the malaria parasite by targeting and inhibiting transmission stages, treating drug-resistant parasites, and resolving the features of severe malaria.

### Viral vectored vaccines

#### Description and mechanism of action

Viral vectors have been extensively utilized in vaccine development for many years [62]. These vaccines employ a harmless virus as a carrier to deliver instructions for producing antigens from the targeted disease-causing virus into cells, thereby triggering a protective immune response against the disease [63]. The virus serves as a delivery vehicle, enabling invasion of the cell and directing it to produce antigens that combat the targeted disease that is being targeted [63]. Viral vector vaccines utilize the cellular machinery of eukaryotic cells to generate antigenic targets, potentially resulting in the production of antigens with their native conformation [62]. Two types of viral vector-based vaccines exist: replicating and non-replicating [64]. Non-replicating viral vector-based vaccines are incapable of generating new viral particles, instead, they solely produce vaccine antigens through the use of replication-deficient viral vectors. On the other hand, replicating vector vaccines produce new viral particles within the cells they infect, which then proceed to invade more new cells, thereby producing the vaccine antigen [64]. Various viral-vectored malaria vaccines have been developed, including poxvirus-vectored malaria vaccines, adenovirus-vectored malaria vaccines, and alphavirus-vectored vaccines [62]. Additionally, emerging viral vectors such as flavivirus vectors, measles virus vectors, and vesicular stomatitis virus vectors offer further opportunities for designing novel malaria vaccine candidates [62]. 

#### Benefits and challenges

Viral vector vaccines offer several advantages compared to conventional subunit vaccines. One key benefit is their ability to induce robust antibody responses as well as cellular reactions that are crucial for eliminating pathogen-infected cells [65]. Unlike many recombinant protein-based vaccines, viral-vector vaccines can generate significant immunogenicity without the need for adjuvants, and they can elicit long-lasting immune responses [62]. Moreover, viral vectors can serve as effective delivery systems for malaria antigens and can be engineered to target specific cells or tissues [62,65]. Replicating viral vectors, in particular, can mimic natural infections, leading to the induction of cytokines and co-stimulatory molecules that possess potent adjuvant effects [65]. 

Furthermore, the development of viral-vectored vaccines often does not require complex process development, as these vaccines typically have a consistent purification process regardless of the specific transgene they express [62]. Additionally, certain viral vectors have the ability to carry multiple genes allowing a single viral-vectored construct to contain antigens from the different stages in the parasite’s life cycle, potentially eliciting broad protective immunity [62]. 

However, a major challenge associated with viral vector vaccines is the scalability of production [63]. Traditional systems that use adherent cells can be difficult to scale up due to the complexity of the process [66]. To address this challenge and mitigate the risks during clinical manufacturing, researchers are now developing suspension cell lines that would enable viral vectors to be grown in larger single-use disposable culture systems and bioreactors [63,66]. 

### Leveraging covid-19 vaccine technology to accelerate the development of malaria vaccine candidates in the pipeline

Over the past few decades, significant efforts and resources have been dedicated to reducing the disease burden and finding a lasting solution to the global health crisis posed by malaria. Despite some progress, malaria remains a major global public health concern worldwide [67]. Advances in scientific and technology research have led to breakthroughs in the field of medical sciences, with a particular focus on reducing the impact of global diseases. One such initiative is the malaria vaccine technology roadmap, first published by the WHO in 2006 and updated comprehensively in 2013 towards meeting specific targets by the year 2030 [68,69]. Based on the results of a pilot implementation program for one of the leading malaria vaccines in the world, which demonstrated modest efficacy against clinical *falciparum* malaria [70] and reduced acute malaria cases by 30% in the first 2 years ^71]^ , the WHO granted clearance for widespread use of RTS, S/AS01, the first malaria vaccine to enter stage 3 clinical trials, among children in sub-Saharan Africa, and other malaria-endemic regions [71]. 

However, the emergence of COVID-19 pandemic caused by the SARS-CoV-2 virus has significantly disrupted progress in finding a solution to the malaria challenge, as global attention has shifted towards addressing the pandemic [72,73]. Nonetheless, one potential positive outcome of this pandemic is the opportunity to leverage vaccine technologies, such as mRNA vaccine technology or the wheat germ cell-free protein synthesis system [74], to expedite the development of a more effective malaria vaccine. In 2022, during the World Malaria Day, the WHO welcomed news that the manufacturers of the Pfizer-BioNTech COVID-19 vaccine intended to utilize mRNA technology for the development of a malaria vaccine [75]. Currently, several malaria vaccines are undergoing trials, although their prospects for higher efficacy than the RTS, S appear limited [76]. Therefore, careful consideration should be given to incorporating novel technologies developed during the fight against COVID-19 into the battle against malaria, as this would offer a comprehensive approach. The actual outcome of these efforts remains to be seen.

## Prospects for future developments in malaria vaccine research

Malaria remains a significant cause of morbidity and mortality worldwide, particularly in tropical and subtropical regions. Although there has been progress in reducing the burden of malaria in recent years, it remains a persistent and deadly disease globally. Africa bears the brunt of the disease, with a large proportion of cases and deaths occurring on the continent.

Advancements in vaccine technology offer hope for success in combating Malaria, despite the challenges that lie ahead. The approval of RTS, S/ASO1 by the WHO for use in children in malaria-endemic areas, such as sub-Saharan Africa, marked a significant milestone in the pursuit of an effective vaccine. This approval has stimulated the development of novel vaccines that aim to surpass the reported efficacy of RTS, S. The pre-erythrocytic vaccine approach has exhibited optimal effectiveness, notably with the use of whole parasite vaccines. However, challenges arise in its widespread application for mass vaccination, particularly when employing irradiated sporozoites. The utilization of genetically attenuated parasites presents a promising avenue for mass deployment, particularly within a multi-stage approach that combines late-arresting genetically attenuated parasites with mRNA vaccines targeting blood-stage candidates. However, careful consideration of diverse molecular adjuvants selection, their advantages and disadvantages are crucial. Transmission-blocking vaccines and pregnancy-related vaccines contribute to the arsenal of malaria prevention.

Eliciting cross-species immune responses, and evidence that any Pf (or Pv) vaccine can induce cross-species protection is a pertinent area of research. This gap in empirical validation necessitates continued clinical trials to evaluate the efficacy of candidate vaccines in providing broader immunity against co-infection with multiple species of malaria parasite. The high disease burden in the global south presents resource constraints in terms of vaccine development and affordability of commercial vaccine supplies.

The COVID-19 pandemic has disrupted research and development efforts to advance the malaria agenda. Even before the emergence of COVID-19, funding for malaria eradication programs, represented by government health expenditure has been decreasing. This decline underscores the need for collaboration among governments in malaria-endemic countries to allocate more national resources to research and development of vaccines for malaria prevention and control. Leveraging malaria vaccine technologies based on COVID-19 vaccines may hold potential for the development of an affordable and effective malaria vaccine. Only time will tell if this proves true.

## Conclusion

Malaria vaccines encompasses two primary categories, namely pre-erythrocytic vaccinations and multistage vaccines, each presenting distinct strategies, advantages, and challenges. Although whole sporozoite vaccines (WSVs) exhibit substantial potential for conferring robust immunity, their widespread production is commercially unfeasible. Conversely, subunit vaccines, while more cost-efficient, demonstrate a decline in immunity over time. The intricacies of host-pathogen interactions, particularly across diverse *Plasmodium* species, underscore the need for more clinical trials to unravel the mechanisms underpinning cross-species immunity.

To advance our capabilities in combating malaria, future research endeavors should prioritize in-depth analyses that comprehend the intricate immunological processes involved in cross-species protection in endemic regions. While innovative strategies in vaccine technology have emerged, addressing challenges such as limited research funding, resource accessibility, and fostering collaborative efforts remains imperative in the multifaceted landscape of malaria prevention and control. Therefore, enhancing the vaccine toolkit and augmenting existing preventive measures are essential components of the global strategy to combat malaria effectively.

## Data Availability

Data sharing does not apply to this article as no new data were created or analyzed in this study.

## Abbreviations

Mosquirix: malaria vaccine RTS
VIMT: vaccines to interrupt malaria transmission
WLA ML4: WHO-listed authority maturity level spz- sporozoites
WS: whole sporozoite
WSV: whole sporozoite vaccines
RASpz: radiation attenuated parasite
GAPSpzV: genetically attenuated parasite sporozoite vaccines
CSP: circumsporozoite protein
TRAP: genetically attenuated parasite sporozoite vaccines
LSA: liver stage antigen
VLP: viral-like particle
DBP: Duffy binding protein
EBA-175: erythrocyte binding antigen 175
TNF: tumor necrosis factor-alpha
RESA: Ring Erythrocyte Surface Antigen
TBVs: transmission-blocking vaccines
mAbs: monoclonal antibodies
MAP: Multiple Antigen Protein
GLURP: glutamate-rich protein
AMA1: apical membrane antigen 1
